# Continuous subcutaneous apomorphine infusion allowing awake deep brain stimulation in a Parkinson’s disease patient

**DOI:** 10.1186/s40734-021-00091-4

**Published:** 2021-04-09

**Authors:** Francesca Spagnolo, Francesco Romeo, Piermassimo Proto, Augusto Maria Rini, Emanuela Leopizzi, Andrea Tedesco, Marco Frizzi, Bruno Passarella

**Affiliations:** 1 Neurological Department, A. Perrino’s Hospital, strada statale 7 per Mesagne, 72100 Brindisi, Italy; 2 Neurosurgical Department, A. Perrino’s Hospital, Brindisi, Italy

**Keywords:** Parkinson’s disease, Deep brain stimulation, Apomorphine, Surgery

## Abstract

**Background:**

Subthalamic Deep Brain Stimulation (DBS) have demonstrated in the last decades to determine an important clinical improvement in advanced and selected Parkinson’s disease (PD) patients. However, only a minority of parkinsonian patients meet the criteria to undergo DBS, and the surgical procedure itself is often stressful, especially for patients experiencing severe OFF state. Subcutaneous Apomorphine continuous administration is suitable as an adjunctive therapy capable of improving a suboptimal DBS result. Here we hypothesize a possible role for subcutaneous apomorphine infusion to alleviate severe OFF state in parkinsonian patients undergoing DBS, thus allowing intraoperative microrecording and patient’s collaboration during clinical testing.

**Case presentation:**

A 68-year-old man, suffering from a very long PD-history, characterized by a severe akinetic status and dramatic non-motor features while in OFF, underwent Subthalamic-DBS keeping a slight but continuous apomorphine infusion (1.8 mg/hour), able to guarantee the right degree of patient’s collaboration without interfering with microelectrode recordings. There were no intra or perioperative complications and after the procedure he experienced a marked clinical benefit, being able to stop apomorphine administration.

**Conclusions:**

Here we described the first Subthalamic DBS procedure performed with a low and stable dopaminergic stimulation guaranteed by subcutaneous Apomorphine continuous infusion. For its rapidity of action and prompt reversibility, apomorphine could be particularly suitable for use during difficult surgical procedures in PD, allowing more therapeutic opportunities for patients who would otherwise be excluded from the DBS option.

**Supplementary Information:**

The online version contains supplementary material available at 10.1186/s40734-021-00091-4.

## Background

among device-aided therapies for advanced Parkinson’s disease (a-PD), Subthalamic Deep Brain Stimulation (STN-DBS) has the advantage to be a one-time procedure generally determining a good, long-lasting, clinical efficacy [1]. Awake STN-DBS surgery generally involves that the anatomic target is further refined by electrophysiological mapping and that intraoperative stimulation defines the therapeutic window of a selected track, trying to avoid unacceptably low side-effect threshold with stimulation. This may lead to less postoperative treatment-induced side effects, compared with surgery performed under general anesthesia [2]. Patients undergoing awake STN-DBS procedures should be alert and cooperative during both the recording and stimulation phases, in order to report any side effect of intraoperative stimulation (for example diplopia, nausea, paresthesia). Speech and oculomotion are also essential aspects to be tested during an awake STN-DBS procedure, as well as stimulation benefit on cardinal PD-motor aspects, and the elicitation of stimulation-induced dyskinesias, indicative of good targeting [3].

The efficacy of continuous subcutaneous apomorphine infusion (APO), and intrajejunal levodopa infusion has been established as well, and with STN-DBS, they are generally considered as options for a-PD. However, the management of a-PD can become complicated, rarely requiring the use of more than one advanced therapy, either at the same time or sequentially [4]. Particularly, APO is suitable as an adjunctive therapy capable of improving a suboptimal STN-DBS result. Apomorphine is a highly potent, non-selective dopamine agonist, with a rapid, short-acting effect, administered subcutaneously, either intermittently or continuously. Low invasiveness, rapidity of action and maneuverability, accompanied by a low cost are the strengths of this therapy, which is also indicated as a substitute for oral dopaminergic medication in PD-patients undergoing abdominal surgery or requiring long-term food and oral medication abstinence [5]. APO in the perioperative DBS period can have a role, as in the case we describe, to enable awake surgery in a subject suffering from a very severe parkinsonian condition.

## Case report

A 68-year-old man, with no significant comorbidities except for a 20-year history of idiopathic PD, presented with worsening parkinsonism, motor fluctuations, peak-dose dyskinesias. Eight years after the diagnosis, unpredictable motor fluctuations, peak-dose dyskinesia and OFF non-motor symptoms consisting of pain and emotional distress, became increasingly difficult to manage with medical therapy. In 2011 he decided to undergo evaluation for STN-DBS in another center, where he was judged ineligible for his poor education. Disappointed, for many years he no longer wanted to consider the option of a reassessment for STN-DBS eligibility. His parkinsonian condition inevitably worsened, alternating very severe blocks with uncontrollable involuntary movements, and forcing family members to continuous day and night care, mainly due to his severe restlessness and pain during OFF periods, with the constant need to be mobilized. He refused the option of levodopa-carbidopa intestinal gel and in 2017 apomorphine subcutaneous pump was assessed in ambulatory setting, showing good benefit and permitting a reduction of oral drugs. In the following months apomorphine infusion was gradually increased from 3.5 mg/hour to 7 mg/hour, with a discrete, but not complete control of OFF-phases. The STN-DBS option was further discussed and finally accepted. Presurgical diagnostic evaluations did not show any contraindications to brain surgery; particularly, compatibly with the level of education, the patient showed no cognitive impairment. His motor score following a levodopa challenge decreased from 104 to 47 on Movement Disorder Society Unified Parkinson’s Disease Rating Scale (MDS-UPDRS; 55% improvement). His OFF period was characterized by an almost complete akinetic status (video_1). We decided to carry out STN-DBS keeping a slight but continuous apomorphine infusion (1.8 mg/hour). A partial motor MDS-UPDRS of assessable items (MDS-UPDRS items: 3.1, 3.2, 3.3b-e, 3.4a-b, 3.5a-b, 3.6a-b, 3.7a-b, 3.8a-b, 3.14, 3.15a-b, 3.16a-b, 3.17a-e, 3.18) was intraoperatively performed under apomorphine infusion before starting stimulation trials, showing a slight reduction of patient’s preoperative OFF sub-score (71/108 versus 80/108), especially due to a slight amelioration of rigidity. However, the main advantage of low-dose apomorphine infusion in our patient was to ensure a good level of cooperation and to limit his discomfort and pain associated with OFF condition.


**Additional file 1: Video 1.** The patient is shown in OFF state, after withdrawal of antiparkinsonian treatment; this condition is characterized by a very serious akinetic state. In the video the patient is instructed to protrude his tongue, to move the right and left hands (Hand grip) and to move up and down the lower limbs (Foot tapping). A rest tremor is evident in his right hand.

The patient underwent bilateral placement of STN-DBS leads (3389, Medtronic, Minneapolis, Minnesota USA). Microelectrode recordings were obtained from both sides. There were no intra or perioperative complications. Four weeks after leads placement, monopolar stimulation of the contacts 2−/11- led to the best clinical response (3.6 V and 3.2 V respectively for left and right STN; frequency 80 Hz; pulse duration 90 usec).

He managed to stop APO in about two months since STN-DBS and to continue his oral and electrical antiparkinsonian therapy with a good motor control. At the 6-months evaluation the patient’s dyskinesias resolved dramatically and his motor MDS-UPDRS (stim ON/drug OFF) was 44.

## Discussion

Dopaminergic deprivation and awake testing are important points in STN-DBS procedures, as they enable intraoperative physiologic target localization and macroelectrode test stimulation [6], improving safety and outcome in STN-DBS for PD [7]. Antiparkinsonian medication has to be interrupted prior to surgery since the trial stimulation is distorted by dopaminergic drugs. However, not all patients tolerate being off medication for such a long time and here we described the first STN-DBS procedure performed with a low and stable dopaminergic stimulation guaranteed by APO. Unfortunately in advanced PD being OFF drug causes much more than just a resurgence of tremor or freezing. Patients with severe OFF are at risk to develop complications linked to dopaminergic drugs withdrawal, including respiratory failure, psychic disorders with agitation, disorientation and, rarely, an acute akinetic attack known as parkinsonism-hyperpyrexia syndrome [8]. Despite rare, these complications frequently require postoperative intensive care unit treatment [9]. Severe neurologic deterioration due to dopaminergic withdrawal in the perioperative period of a STN-DBS procedure is estimated to be around 3.2% [10]. To avoid these complications, some centers prefer to perform STN-DBS surgery under general anesthesia, sacrificing important clinical and neurophysiological informations. However, data from a single-center [7] and other reports suggest that apomorphine could be helpful during awake STN-DBS to obtain the right degree of collaboration by the patient, without interfering with micro-recording and relieving patients’ discomfort [7, 11, 12]. To our knowledge this is the first time that APO was efficaciously used during STN-DBS, in a patient experiencing very severe OFF. Slotty et al. [7] reported using APO in the perioperative management of PD-patients, stopping it 2 h prior to surgery and restarting it after electrode placement. In their 5-year-single center experience, authors reported no significant side effects and a reduced postoperative neurologic deterioration. Martin de Campos utilized three subcutaneous 2 mg apomorphine injections in the course of a PD-patient STN-DBS procedure. In our procedure, we decided to keep APO throughout all the procedure, at a dosage lower than the patient’s usual one, enabling his collaboration, electrophysiological STN recording and clinical assessment. Apomorphine injection has a quick onset of effect (typically within 4–12 min) with a rapid clearance half-life and a mean duration of anti-parkinsonian action lasting about 45–60 min [13]. The choice of keeping a low and stable dopaminergic stimulation guaranteed by APO continuous infusion rather than intermittent injections was motivated by the desire to avoid to greatly interfere with intraoperative clinical assessment as well as to prevent a severe OFF state with intolerable symptoms during the surgical procedure, which would have dramatically complicate patient’s cooperation. A “controlled relief” of motor and non-motor off symptoms was reached with a low and constant dopaminergic continuous stimulation, allowing a patient-specific tailored approach to implantation. We believed that the brief half-life and the prompt reversibility of intermittent apomorphine injections could not suit for our intent.

Administration of apomorphine does not result in a true restoration of the non-linear properties of STN firing, however few studies showed that clinically effective doses of apomorphine partially restore STN activity, leading to a decrease in entropy measured in the inter-spike intervals of Subthalamic neurons of PD patients [14] and significantly decreasing the overall firing rates of GPi neurons and of STN neurons in dyskinetic PD-patients [15]. In both the mentioned studies apomorphine was administered at a clinically significant dosages (4.7 ± 0.8 and 2.5–8 mg, respectively), higher than the one we used to relief our patient’s OFF-state. This is probably the reason why in our case a typical STN recording was obtained from both nuclei, despite apomorphine infusion.

Once again, our report stresses the understanding that a-PD can require a very complex management, sometimes adding the benefit of more than one device-aided therapy. We believe that apomorphine can allow more therapeutic opportunities for PD-patients who would otherwise be excluded from surgery. Its rapidity of action and its prompt reversibility makes apomorphine particularly suitable for use during surgical procedures in PD-patients.

## Data Availability

Not applicable.
